# Extraction of Triterpenic Acids and Phytosterols from Apple Pomace with Supercritical Carbon Dioxide: Impact of Process Parameters, Modelling of Kinetics, and Scaling-Up Study

**DOI:** 10.3390/molecules23112790

**Published:** 2018-10-27

**Authors:** Łukasz Woźniak, Anna Szakiel, Cezary Pączkowski, Krystian Marszałek, Sylwia Skąpska, Hanna Kowalska, Renata Jędrzejczak

**Affiliations:** 1 Department of Fruit and Vegetable Product Technology, Institute of Agricultural and Food Biotechnology, 36 Rakowiecka Street, 02532 Warsaw, Poland; krystian.marszalek@ibprs.pl (K.M.); sylwia.skapska@ibprs.pl (S.S.); 2 Department of Plant Biochemistry, Faculty of Biology, University of Warsaw, 1 Miecznikowa Street, 02096 Warsaw, Poland; szakal@biol.uw.edu.pl (A.S.); myhacp@biol.uw.edu.pl (C.P.); 3 Department of Food Engineering and Process Management, Faculty of Food Sciences, Warsaw University of Life Sciences, 161/59c Nowoursynowska Street, 02787 Warsaw, Poland; hanna_kowalska@sggw.pl; 4 Department of Food Analysis, Institute of Agricultural and Food Biotechnology, 36 Rakowiecka Street, 02532 Warsaw, Poland; renata.jedrzejczak@ibprs.pl

**Keywords:** β-sitosterol, broken plus intact cell model, *Malus domestica*, supercritical fluid extraction, ursolic acid

## Abstract

Apple pomace, a byproduct of juice production, is a rich source of bioactive compounds and nutrients. Supercritical fluid extraction was proposed as a method for a fast and selective extraction of hydrophobic compounds with a pharmaceutical potential from this matrix. Chromatographic analysis showed that the pomace contained significant amounts of such substances, the most abundant of them were ursolic acid, oleanolic acid, and β-sitosterol. The solubility was chosen as a primary factor for a selection of the extraction conditions; the best results were acquired for a temperature of 80 °C and a pressure of 30 MPa. The equation proposed by Chrastil was applied for the description of the impact of the process parameters on the solubility of the analytes; the obtained values of coefficients of determination were satisfactory, despite the fact that the equation was developed for binary systems. The extraction curves obtained during the experiments were used for the description of the process kinetics using the Broken plus Intact Cell model. The impact of the temperature, pressure, and flow rate of carbon dioxide on the mass transfer phenomena was investigated. The data obtained allowed the prediction of the extraction curve for the process conducted on the larger scale.

## 1. Introduction

Supercritical fluid extraction (SFE) is an alternative for processes of extraction using organic solvents of a low polarity. Carbon dioxide, the solvent of choice in the vast majority of applications, can be more advantageous than the organic solvents due to its properties, such as its low cost, chemical inertia, and minor impact on human health and the environment. The main constraints of this method are connected with a limited range of compounds that can be extracted with pure CO_2_ and a high cost of the equipment. The commercial uses of SFE in formulation of food products include: production of extracts from hop and spices, decaffeination of coffee and tea, as well as removal of fat from various products [1]. Supercritical fluids have been also implemented for processing of food industry waste products; these applications include isolation of nutrients, biodiesel production, and hydrolysis of lignocellulosic materials [2].

The features of SFE, such as higher selectivity of extraction and reduction of use of organic solvents, are especially beneficial during the purification of high-value bioactive compounds of a natural origin. A recent review by de Melo et al. [3] reports the extraction of more than 300 plant species with supercritical carbon dioxide. These experiments were conducted using various parts of plants, but most of them concerned seeds and leaves, while processed plant material was used only in approximately 5% of the studies. The range of compounds isolated using SFE included phenolic compounds, triglycerides, terpenoids, and fat-soluble vitamins among others. The authors of the review noticed that the vast majority of SFE studies had concerned only production of the extracts and their characterization. Despite the fact that knowledge about the kinetics of the extraction is essential for a practical implementation of the process, in most of the studies only a selection of optimal conditions was performed.

Apples (*Malus domestica* Borkh.) are one of the most consumed fruits, with a yearly production of approximately 80 million tonnes [4]. Apart from their sensory quality, the apples are also a source of compounds exhibiting high pharmacological potential as has been confirmed by several research teams [5,6,7]. Two the most important groups of low-polarity bioactive compounds found in apple are triterpenic acids and phytosterols. The triterpenic acids are constituents of cuticle waxes covering the aerial parts of plants. They received much attention in recent years due to their wide spectrum of pharmacological activities. Ursolic acid is the most abundant of the triterpenic acids in apples; the literature reports that it exhibits some interesting health-promoting properties and could even be implemented in the therapy of several types of cancer [8]. Phytosterols are important structural components of plant membranes; this group of compounds is known mostly for their cholesterol-lowering potential. Clinical studies showed that the daily intake of 1–3 g of phytosterols and their derivatives—phytostanols—can significantly reduce serum cholesterol levels and in consequence decrease the risk of cardiovascular diseases [9].

Thus far, experiments on the extraction of bioactive compounds from apple with supercritical carbon dioxide focused on the other groups of compounds. Massias et al. [10] used a mixture of ethanol and CO_2_ at 25 MPa and 50 °C for isolation of phenolic compounds. Li et al. [11] compared the isolation of lipids during extraction with hexane and carbon dioxide at 35 MPa and 50 °C; however their work concentrated mainly on the isolation of hydrocarbons. Additionally, two research groups investigated the possibility of using supercritical CO_2_ for the removal and analysis of pesticides and fungicides from apple products [12,13]. The possibility of using SFE to isolate triterpenic acids and phytosterols has been already proved in numerous publications; the literature presents data for, inter alia, birch [14], eucalyptus [15], grape [16], and marigold [17].

This paper is a report on experiments on extraction of triterpenic acids and phytosterols from apple pomace with supercritical carbon dioxide. The experiments included the evaluation of the bioactive compounds content in the matrix, the investigation of the impact of the process parameters on the solubility of the analytes, the modelling of the process kinetics, and the verification of the data acquired during the scaling-up experiment. The ultimate goal of the study was to gather the data required for industrial implementation of the process. According to the authors’ best knowledge this is the first study focusing on the isolation of triterpenic acids and phytosterols from the apple pomace.

## 2. Results

### 2.1. Apple Pomace as a Source of Triterpenoids

**Figure 1:**
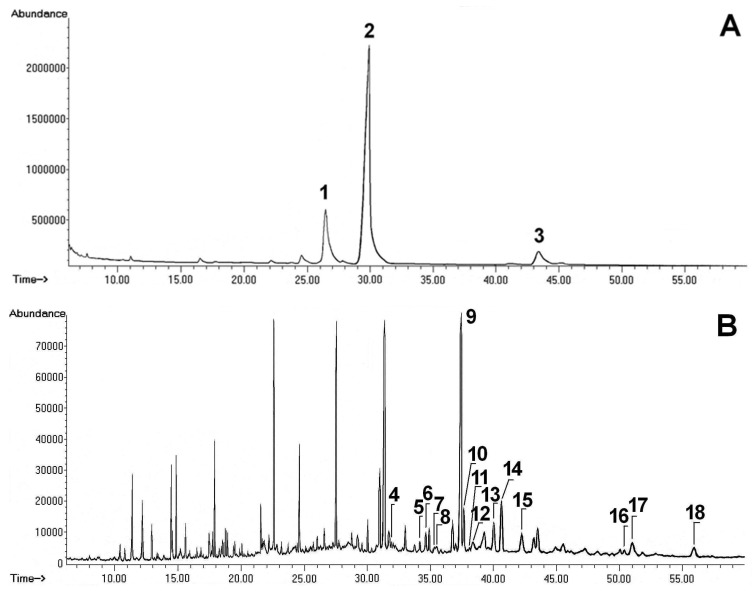
Representative GC-FID chromatograms of the fraction containing methyl esters of triterpene acids (**A**) and the fraction containing sterols and neutral triterpenes (**B**) obtained from apple pomace. Peaks are numbered according to the retention times: (**1**) oleanolic acid (methyl ester), (**2**) ursolic acid (methyl ester), (**3**) pomolic acid (methyl ester), (**4**) cholesterol, (**5**) cholesta-3,5-dien-7-one, (**6**) campesterol, (**7**) cholest-4-ene-3,6-dione, (**8**) stigmasterol, (**9**) sitosterol, (**10**) isofucosterol, (**11**) cycloartanol, (**12**) β-amyrin, (**13**) α-amyrin, (**14**) stigmasta-3,5-dien-7-one, (**15**) sitostenone, (**16**) ursolic aldehyde, (**17**) erythrodiol, (**18**) uvaol. Peaks not numbered are associated with aliphatic or phenolic compounds.

**Figure 2:**
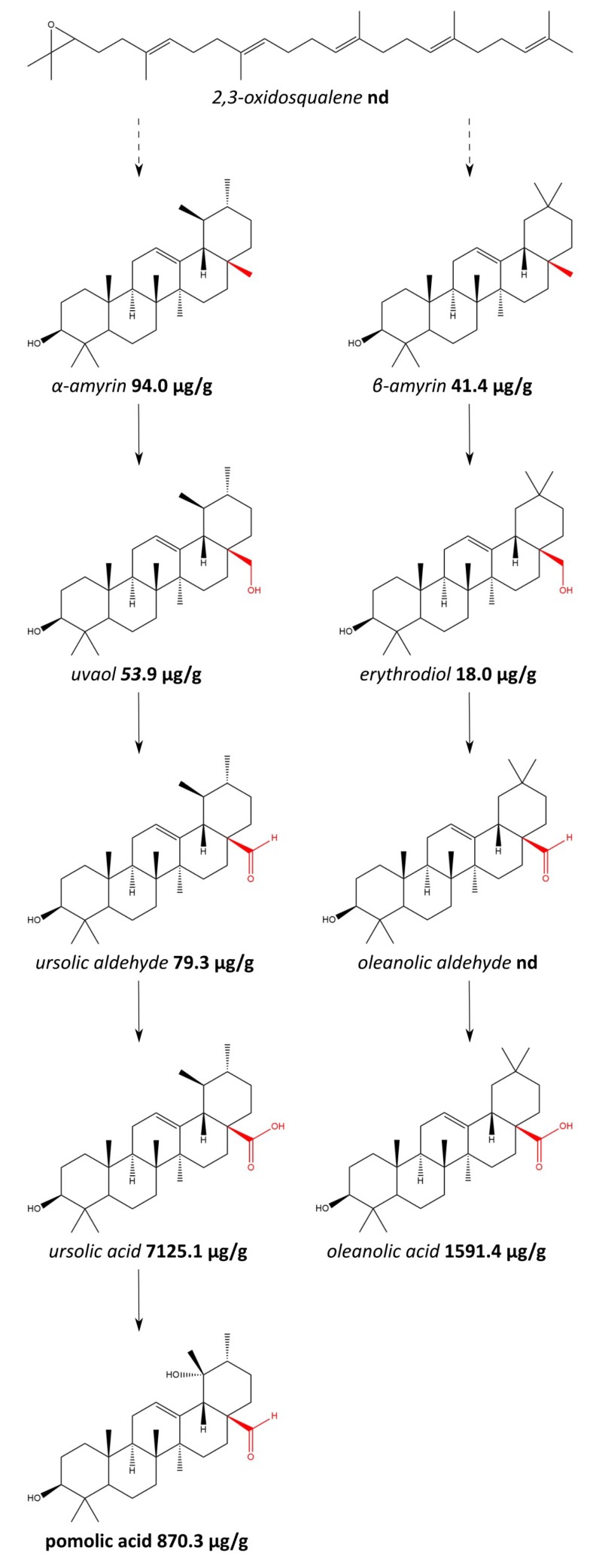
Triterpenic acids and intermediates of their biosynthesis; the results of quantification in the apple pomace. Abbr.: nd—not detected.

**Figure 3:**
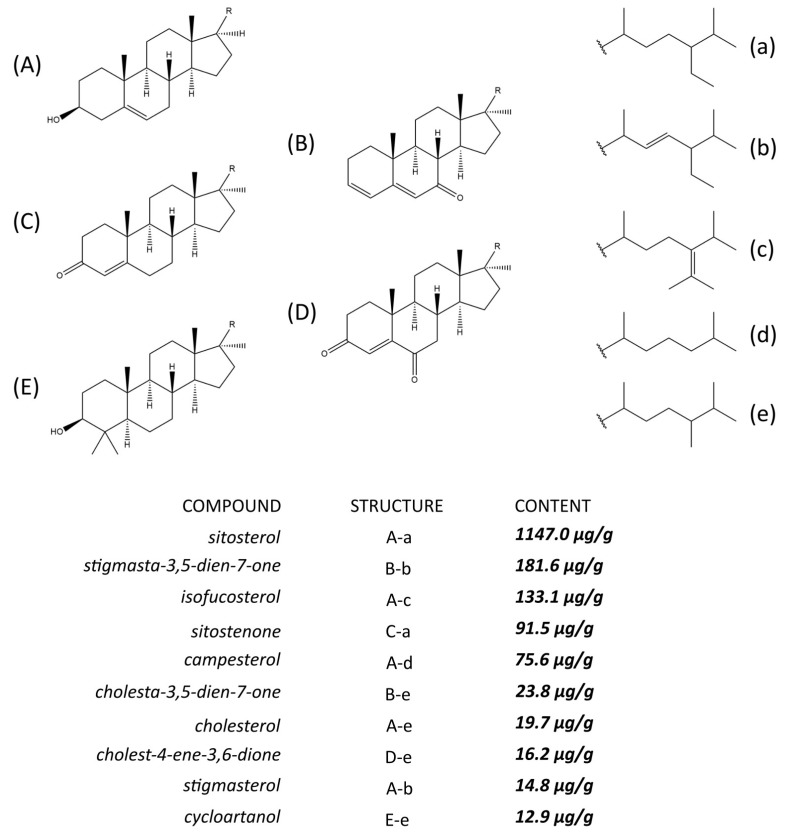
Sterols and their derivatives present in the apple pomace.

The experiments proved that investigated apple pomace was a rich source of lipophilic compounds with pharmacological potential. An analysis of Soxhlet extraction of the plant material provided data on the total amount of compounds of interest in the pomace. The chromatograms of are presented in Figure 1. The content of triterpenic acids and intermediates of their biosynthesis is presented in Figure 2. The amount of sterols and their derivatives is presented in Figure 3. The analyses showed that the pomace did not contain significant amount of low-polarity ester fraction.

The total triterpenic content was 9.586 mg/g of pomace. Ursolic acid was the most abundant analyte and consisted of 7.125 mg/g (approx. 75% of the total). Significant amounts of intermediates of the triterpenic acids synthesis were also observed in the pomace (0.287 mg/g). The literature provides only the data on the content of triterpenic acids in fresh apples or apple peel; however the results can be compared with this work. Andre et al. [18] investigated content of triterpenic acids in 109 varieties of apple. The amount of triterpenes was greatly affected by the cultivar; the most abundant compounds were ursolic and oleanolic acid, their content in fresh fruit was 44.73–322 μg/g and 47.2–838 μg/g, respectively. Frighetto et al. [19] performed an analysis of the ursolic acid content in four apple cultivars; however, the results were presented considering the area, not the mass, of the apples. The ursolic acid content was 0.21–0.82 mg/cm^2^, i.e., 11.7–65.0 mg per apple. The triterpenes content was also investigated by Grigoras et al. [20] although only a qualitative and semi-quantitative analysis was conducted. The most abundant triterpenes were ursolic and oleanolic acid, however numerous other compounds were identified including pomolic acid, betulinic acid, and *trans*-*p*-coumaryloxy derivatives of the triterpenes.

The total content of sterols and their derivatives in the pomace was 1.716 mg/g. Similarly to the previous group, one of the compounds (β-sitosterol) constituted most of the content. Lower amounts of other sterols as well as their oxidized forms were also found. The sterol content in the apples was previously investigated by, among others, two research teams from Scandinavia. Normén et al. [21] reported that one gram of fresh apple contained 130 μg of sterols, while Piironen et al. [22] reported 1.5 mg of sterols per gram of apple dry weight; these values are similar on a dry mass basis. β-sitosterol was the most abundant sterol in both studies.

Apple pomace is a convenient material for extraction: its low cost, status of the waste product, and high content of bioactive compounds make it more appealing material than whole apples. On the other hand, the composition of the pomace depends on numerous factors, including: the technology used in production, the cultivars used, weather conditions, and harvesting time; therefore, the composition of product obtained shows significant variations. The chemical nature and biological role of the compounds can impact their partition between juice and pomace. Triterpenic acids are localized mainly in the cuticular waxes protecting the fruit from stress factors [7]; therefore, the vast majority of them remain in the pomace. Meanwhile, the sterols are present in all cells as a natural constituents of lipid bilayer [11], thus in the case of cloudy juices only part of them will be found in the pomace.

### 2.2. Selection of the Extraction Conditions

The two analytes that were the most abundant in the groups of compounds studied (ursolic acid and β-sitosterol) were chosen for a selection of the extraction conditions and analysis of results of further experiments. The process parameters used by other research teams for isolation of triterpenic acids and plant sterols were in a very wide range; according to a review by de Melo et al. [23] the operating conditions were: temperatures of 40–90 °C, pressures of 10–50 MPa, and the addition of up to 5% of cosolvent. Nine sets of parameters were selected for the experiments: combinations of three temperatures (40, 60, and 80 °C) and three pressures (10, 20, and 30 MPa). The study did not included use of cosolvents during the extraction because of technical limitations of the extractor used. Additionally, the leftovers of organic solvents would remain in the pomace and thus restrain its use, for example, as a fodder. The solubilities obtained during the experiments are listed in Table 1.

The solubility of analytes was significantly influenced by both parameters. In general, the solubilities were bigger for higher temperatures and pressures. The solubility of ursolic acid was significantly lower than the solubility of β-sitosterol; in some of the tested parameters it was too low for the quantification. It must be noted that, due to the methodological approach, the measured solubilities are impacted by presence of other constituents of the matrix. Therefore, the results obtained may vary from the values obtained in ursolic acid–CO_2_ and β-sitosterol–CO_2_ binary systems.

The selectivity of the extraction can be expressed as a fraction of ursolic acid and β-sitosterol in the extracts obtained. The selectivity of the extraction was generally the highest for the conditions with biggest solubility, possibly due to full extraction of low-weight hydrophobic compounds with all the parameters tested. During the tests, several ratios of matrix mass and supercritical fluid volume were tested to ensure obtaining the maximal solubility strongly affecting the fraction of tested analytes in the extracts.

The impact of the carbon dioxide parameters on the solubility was analyzed using an equation proposed by Chrastil (Equation (1)) [24]:

(1) $$c={d_{f}}^{k}\times exp\left(\frac{a}{T}+b\right)$$

**Figure 4:**
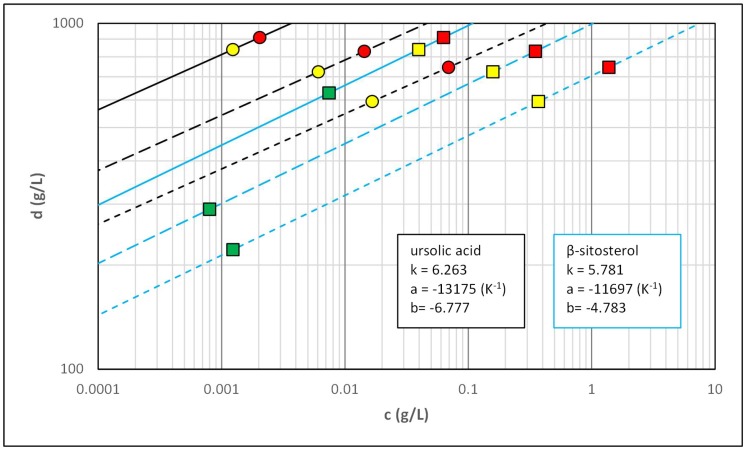
The application of Chrastil’s equation for the description of the solubility of ursolic acid and β-sitosterol in supercritical carbon dioxide. Symbols: black lines and circles—ursolic acid, blue lines and squares—β-sitosterol, solid lines—40 °C, long-dashed lines—60 °C, short-dashed lines—80 °C, green markers—10 MPa, yellow markers—20 MPa, red markers—30 MPa.

The densities of carbon dioxide for the experimental points were calculated using online tool based on equation of state presented by Span and Wagner [25]. The graphical presentation of the results on log-log plot is presented in Figure 4. The parameters of the equation can be connected with properties of the solvation system: *k* is an association number, *a* is a sum of heat of evaporation and solvation divided by gas constant, while *b* is a function of molecular masses of the solvent and the solute.

Despite the fact that Chrastil’s equation was created for the description of solubility of pure compounds in binary systems, such approach proved to be suitable for the description of the solubilities of minor constituents in complex matrix. The mean weighted differences between obtained data and Chrastil’s model were 8.8% and 4.2% for ursolic acid and β-sitosterol, respectively. The equation could also be used for predicting the solubility of ursolic acid and β-sitosterol in conditions that were not investigated. The values of the parameters of the equation are similar to those presented in the literature data for other groups of compounds [24].

### 2.3. Mathematical Modelling of the Process

Three sets of process parameters characterized by the highest solubility of analytes (80 °C/30 MPa, 80 °C/20 MPa, and 60 °C/30 MPa) were selected for the experiments. Additionally, three flow rates were tested at 80 °C/30 MPa to evaluate an impact of this parameter on the results obtained. The features of the fixed bed were identical for all extraction runs; the characteristics of the bed are listed in Table 2.

The extracts obtained during the experiments were analyzed for the content of ursolic acid and β-sitosterol. Extraction times were paired with cumulative yields to create extraction curves, which were subsequently described by Broken plus Intact Cell (BIC) model. The mass transfer coefficients and fraction of less accessible solute obtained during the fitting of experimental data are listed in Table 3.

The values of the mass transfer coefficients were affected by several factors including the structure of the analytes, flow rate, and properties of supercritical fluid. The coefficients for the easily accessible solute were two orders of magnitude higher than those of the analytes placed inside the intact cells. The values obtained are similar to the values reported in the literature [3]. The mass transfer coefficients for ursolic acid were higher than those for β-sitosterol. The molecular mass of β-sitosterol is lower, however the presence of the hydrocarbon side chain and subsequent increase of the molecular diameter possibly plays a more significant role.

**Figure 5:**
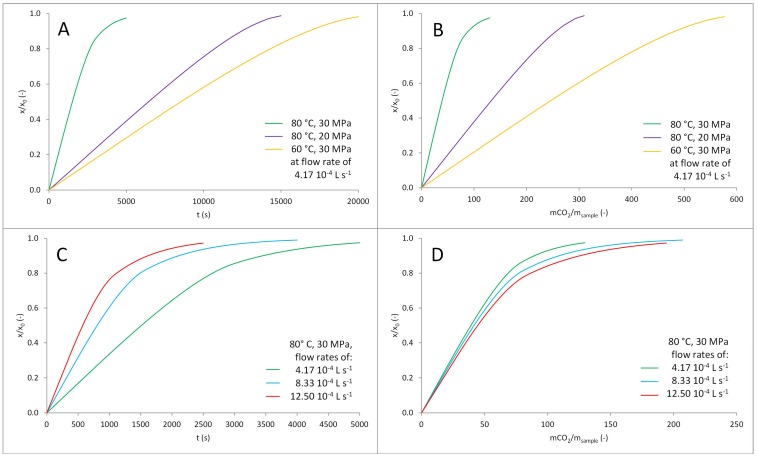
The extraction curves for the isolation of ursolic acid. The impact of temperature and pressure as a function of time (**A**) and mass of carbon dioxide per mass of sample (**B**). The impact of flow rate as a function of time (**C**) and mass of carbon dioxide per mass of sample (**D**).

The extraction curves for the ursolic acid are presented in Figure 5. The authors decided to present the curves in duplicate: as a function of the time and as a function of supercritical fluid used. Such an approach is a result of two possible methods of optimization process: reduction of the extraction time or minimization of amount of carbon dioxide used.

The rate of extraction was significantly affected by pressure and temperature of carbon dioxide; the fastest extraction was observed for 80 °C and 30 MPa. The observed differences were mainly a result of differences in solubility of analytes; the impact of mass transfer coefficients can be, in this case, neglected. The rate of extraction was increasing with flow rate. The time of the process could be significantly shortened by using higher flow rate, although it was connected with a slight increase of mass of carbon dioxide required for the extraction.

### 2.4. Scaling-Up of the Process

The experiment on a larger scale (500 mL vessel) was performed using temperature and pressure characterized by highest extraction rate (80 °C, 30 MPa). The volumetric flow rate was increased proportionally to the vessel volume; although, due to technical restrictions it was only possible to test the smallest flow rate (8.69 10^−3^ L/s).

The scaling up process was performed basing on the criteria presented by Perrut [26]. The mass of spent carbon dioxide per mass of matrix as well as ratio of flow rate and mass of the matrix were kept constant compared to 24 mL vessel. The packed bed was prepared in a manner ensuring the same porosity and specific surface area as on a smaller scale.

Additionally, due to the geometry of the vessels, it was possible to fix the dimensionless number connected with mass transfer. The empirical correlations describing mass transfer coefficients are based on Reynolds and Schmidt numbers (Equations (2)–(3)) [27]. The application of carbon dioxide under the same conditions as well as identical flow speed (*u*) will ensure that mass transfer coefficient will be the same after scaling-up the extraction.

(2) $$Re=\frac{d_{f}\times u\times d_{p}}{\mu }$$

(3) $$Sc=\frac{\mu }{d_{f}\times D}$$

**Figure 6:**
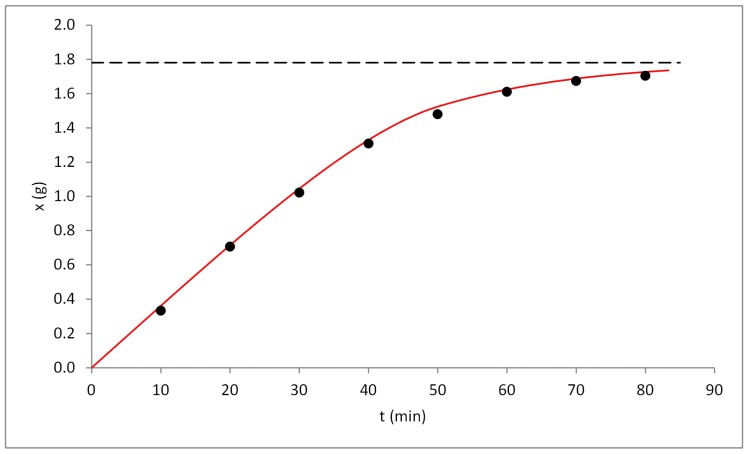
The comparison of the yield of ursolic acid obtained during extraction in a 500-mL vessel (black dots) and the extraction curve predicted using Broken plus Intact Cell (BIC) model (red line).

The comparison between the empirical data from the extraction process and the yield predicted by kinetic modelling is presented in Figure 6. The proposed model proved to be a suitable method for prediction of the extraction curves during increasing the scale of the process. The mean relative differences between the model and experimental data were 1.82% and 4.73% for ursolic acid and β-sitosterol, respectively.

## 3. Conclusions

Supercritical fluid extraction with carbon dioxide proved to be an interesting alternative for organic solvents in the isolation of triterpenic acids and sterols from apple pomace. The results obtained were affected by many factors including the temperature and pressure of carbon dioxide and the flow rate. The Chrastil’s equation was used to describe the impact of the parameters on the solubility of ursolic acid and β-sitosterol, while the BIC model provided information about mass transfer coefficients and the fraction of less accessible solute. The data obtained were used to predict the outcome of a larger scale experiment.

The process was modelled using two of eighteen compounds identified in the pomace. However, the kinetics of the extraction of some of the unexamined compounds could be predicted. The kinetics of the extraction of structural isomers would be very similar (e.g. oleanolic acid and ursolic acid). The polarity of the analytes would possibly be the most important factor affecting their solubility and, as a consequence, the possibility of their isolation with supercritical carbon dioxide.

The paper shows the possibility of the industrial-scale application of supercritical fluid extraction of high value compounds from apple pomace. The possibility of predicting the course of higher scale extraction using BIC model was reported. Moreover, the possibility of utilization of Chrastil’s equation and BIC model for the description of extraction of the minor constituents of the matrix was reported.

## 4. Materials and methods

### 4.1. Materials

The apple pomace was acquired from a local juice producer; it was obtained on a basket press using a mixture of apple cultivars. The pomace was subsequently dried in a laboratory convection dryer (Warsaw University of Life Sciences, Warsaw, Poland) with a forced airflow velocity of 2 m/s at a temperature of 55 ± 2 °C. The samples were placed on perforated shelves in an approx. 5–6 cm layer and dried for a total of 8 h. The content of dry weight in pomace after drying was 88.7 ± 0.1%. The pomace was then ground using RAS mill (Romer Labs, Newark, DE, USA). The average diameter of particles obtained during the milling was approximated using an Olympus CX40 phase-contrast microscope (Olympus, Tokyo, Japan). The portions of the powder were put on a Thoma chamber and the size of over one hundred particles was measured.

Analytical standards of ursolic acid and β-sitosterol were acquired from Sigma-Aldrich (Saint Louis, MO, USA). The methanol used for chromatographic analyses was HPLC grade and was purchased from POCh (Gliwice, Poland). Other chemicals were of analytical grade and came from various suppliers. Technical grade carbon dioxide was acquired from Multax (Zielonki-Parcela, Poland).

### 4.2. Extraction Procedures

The process of extraction with carbon dioxide was carried out in Spe-ed SFE-4 apparatus (Applied Separations, Allentown, PA, USA). During the experiments two extraction chamber sizes were used: the majority of the experiments was performed with a 24-mL vessel, while during the scaling-up experiments, a 500-mL vessel was used. Portions of milled pomace were placed inside the chamber for 30 min prior to extraction to achieve the expected temperature. The sample was subsequently rinsed with the carbon dioxide using pre-selected values of temperature, pressure, and flow rate. During each run several fractions of extract were collected for the preestablished periods of time.

The full extraction of lipophilic bioactive compounds from the pomace was conducted using Soxhlet apparatus. Two samples of pomace (5.00 g each) were extracted for 8 h using 250 mL of diethyl ether.

### 4.3. Determination of Solubility in Supercritical Carbon Dioxide

The solubility of ursolic acid and β-sitosterol was chosen as a primary factor in selection of temperature and pressure for the extraction. For the experimental determination of the solubility, the above-mentioned (Section 4.2.) extraction apparatus was used with 500-mL vessel. The vessel was packed with the milled pomace and, after thermostating, filled with carbon dioxide under chosen parameters. The vessel was left for 120 min in order to achieve maximal possible solubilization of analytes in supercritical CO_2_. Subsequently, the flow was induced and extract from the first 25 mL of carbon dioxide was captured. It was assumed that solubility of ursolic acid and β-sitosterol is equal to their concentration in supercritical fluid leaving the vessel.

### 4.4. Analytical Methods

Three methods of an analysis were used during the research. The content of triterpenoids in pomace was analyzed using the GC-MS-FID method. The content of ursolic acid and β-sitosterol in the SFE extracts was analyzed using HPLC methods.

#### 4.4.1. Identification of Compounds Present in the Pomace

The identification and quantification of the triterpenoids present in the pomace was performed using Soxhlet extract in accordance with the procedure presented by Pensec et al. [28]. The first stage consisted separating groups of the compounds using preparative thin layer chromatography; during the partition, glass plates covered with 60G silica gel were eluted using a mixture of chloroform and methanol (97:3 *v/v*). Three fractions were collected after separation: triterpenic acids (*R_f_* 0.2–0.3), free sterols and neutral triterpenes (*R_f_* 0.3–0.9), and low-polarity esters of triterpenes and sterols (*R_f_* 0.9–1.0).

The ester fraction was dissolved in 10% (*m/v*) sodium hydroxide in 80% (*v/v*) methanol and hydrolyzed for 3 h at 80 °C. The reaction mixture was neutralized with acetic acid and extracted with diethyl ether. The compounds obtained were again separated using aforementioned TLC method. The triterpenic acid fraction was methylated prior to analysis to increase its volatility and enhance chromatographic separation. The samples were dissolved in an ether solution of diazomethane and incubated for 24 h at 2 °C. The fraction of free sterols and neutral triterpenes was analyzed without any pretreatment.

An Agilent Technologies 7890A gas chromatograph (Perlan Technologies, Warsaw, Poland) equipped with a 5975C mass spectrometric detector was used for qualitative and quantitative analyses. GC separation was performed on HP-5MS UI, 30 m × 0.25 mm, 0.25 μm film column (Agilent Technologies, Santa Clara, CA, USA) either under isothermal conditions at 280 °C (triterpene acids methyl esters) or in the temperature program: initial temperature of 160 °C held for 2 min, then increased to 280 °C at 5 °C per min and final temperature of 280 °C held for a further 44 min (steroids and neutral triterpenes) with a constant helium flow rate of 1 mL/min. The samples were dissolved in a mixture of diethyl ether and methanol (5:1, *v/v*) and injected into column using a 1:10 split. Two modes of detection were used simultaneously. The analytes were identified with mass spectrometer (MS) by comparing their mass spectra with spectral libraries and the results of earlier experiments (the energy of ionization was 70 eV, while the ions were analyzed in an *m/z* range of 33–500). A quantitative analysis of all identified compounds was conducted with a flame ionization detector (FID; part of 7890A chromatograph) using the external standard method based on calibration curves determined for the compounds belonging to the representative triterpenoid classes.

#### 4.4.2. Quantification of Ursolic Acid

The ursolic acid content in the extracts was determined using the HPLC-UV method presented by Woźniak et al. [29]. The extracts were dissolved in methanol and purified on Supelclean LC-SAX cartridges (Sigma-Aldrich). An analysis of 25 μL samples was conducted on Zorbax Eclipse PAH 5 μm, 4.6 x 250 mm column (Agilent Technologies). The column was thermostated at 30 °C and eluted isocratically with a mixture of methanol and phosphate-citrate pH 3.0 buffer (9:1, *v/v*) at a flow rate of 0.6 mL/min. The content of ursolic acid was quantified at a wavelength of 205 nm.

#### 4.4.3. Quantification of β-Sitosterol

The quantification of β-sitosterol was performed using HPLC method of Borkovcová et al. [30]. The extracts were dissolved in methanol and analyzed (10 μL) using Sunfire C8, 5 μm, 4.6 × 250 mm column (Waters, Milford, MA, USA). The column was thermostated at 35 °C and eluted with a 0.7 mL/min flow of a mixture of methanol and water (95:5, *v/v*). The β-sitosterol content was analyzed at a wavelength of 205 nm.

### 4.5. Modelling of Extraction

The kinetics of the extraction process was described using the Broken plus Intact Cell (BIC) model presented by Sovová [31] for SFE of plant material. The model is based on the assumption that particles made of several plant cells are formed during grinding of the tissue. The structure of the cells on the surface of the particles is disrupted, therefore allowing easy access to the solute. The cells in the core of the particles are intact, therefore extraction of the solute from them is slower and is limited by the diffusion inside the particle. The extraction curve is divided into three stages on the basis of mass transfer phenomena occurring. The equations of the fragments of the curve (4–6) are presented below; the wider description of the model assumptions and its kinetics can be found in excellent reviews by Huang et al. [32] and Oliveira et al. [27]. The stages of extraction are:

- CER (Constant Extraction Rate)
  (4) $$x=y^{*}\times q_{m}\times t\times \left[1-exp\left(-Z\right)\right]\;$$
- FER (Falling Extraction Rate)
  (5) $$\;x=y^{*}\times q_{m}\times \left[t-t_{CER}\times exp\left(Z_{w}-Z\right)\right]$$
- DC (Diffusion-controlled)
  (6) $$x=x_{0}-\frac{y^{*}}{W}\times ln\left\{1+\left[exp\left(\frac{W\times x_{0}}{y^{*}}\right)-1\right]\times exp\left[W\times q_{m}\times \left(t_{CER}-t\right)\right]\times r\right\}$$

The formulas for calculation of end-points of the extraction stages and dimensionless parameters used in abovementioned equations are presented in Equations (7)–(11).

(7) $$t_{CER}=\frac{x_{0}\times \left(1-r\right)}{y^{*}\times Z\times q_{m}}\;$$

(8) $$\;t_{FER}=t_{CER}+\frac{1}{W\times q_{m}}\times ln\left[r+\left(1-r\right)\times exp\left(\frac{W\times x_{0}}{y^{*}}\right)\right]\;$$

(9) $$\;Z=\frac{k_{f}\times a_{0}\times d_{f}}{q_{m}\times \left(1-\varepsilon \right)\times d_{s}}\;$$

(10) $$\;W=\frac{k_{s}\times a_{0}}{q_{m}\times \left(1-\varepsilon \right)}\;$$

(11) $$\;Z_{W}=\frac{Z\times y^{*}}{W\times x_{0}}\times ln\left[\frac{exp\left[W\times q_{m}\times \left(t-t_{CER}\right)\right]-r}{1-r}\right]\;$$

During the model fitting the values of a fraction of less accessible solute (*r*) and mass transfer coefficients (*k_s_* and *k_f_*) are optimized to minimize the differences between model equation and experimental data.

## Figures and Tables

**Table 1:** The impact of temperature and pressure on solubility of ursolic acid and β-sitosterol in supercritical carbon dioxide.

Temperature (°C)	Pressure (MPa)	Ursolic Acid Solubility (mg/L)	β-Sitosterol Solubility (mg/L)
40	10	nd	7.02
40	20	1.12	42.1
40	30	2.43	62.1
60	10	nd	0.82
60	20	6.69	147
60	30	12.8	374
80	10	nd	1.24
80	20	16.4	352
80	30	72.3	1420

nd—not detected.

**Table 2:** The features of the fixed bed using during the experiments.

Parameter	Value
volume (*V)*	24.0 mL
mass of sample (*m*)	12.00 g
density of sample (*d_s_*)	825 g/L
mean diameter of particles (*d_p_*)	9.4 10^−5^ m
bed porosity (*ε*)	0.394
specific surface area (*a_0_*)	3.87 10^4^ m^−1^

**Table 3:** The kinetic parameters of broken plus intact cell model obtained for tested process parameters.

Parameters		Ursolic Acid		β-Sitosterol	
	r (-)	k_s_ (m/s)	k_f_ (m/s)	k_s_ (m/s)	k_f_ (m/s)
80 °C, 30 MPa, 4.17 10^−4^ L/s	0.341	1.49 10^−8^	1.43 10^−6^	1.21 10^−8^	1.31 10^−6^
80 °C, 30 MPa, 8.33 10^−4^ L/s		1.98 10^−8^	2.12 10^−6^	1.74 10^−8^	1.88 10^−6^
80 °C, 30 MPa, 12.50 10^−4^ L/s		2.40 10^−8^	2.51 10^−6^	2.05 10^−8^	2.22 10^−6^
80 °C, 20 MPa, 4.17 10^−4^ L/s		1.92 10^−8^	1.75 10^−6^	1.68 10^−8^	1.56 10^−6^
60 °C, 30 MPa, 4.17 10^−4^ L/s		1.20 10^−8^	1.32 10^−6^	0.98 10^−8^	1.13 10^−6^

## Nomenclature

*a*: coefficient of Chrastil’s equation (K^−1^)
*a_0_*: specific surface area (m^−1^)
*b*: coefficient of Chrastil’s equation (-)
*c*: solubility (g/L)
*d_f_*: density of carbon dioxide (g/L)
*d_p_*: mean diameter of particle (m)
*d_s_*: density of solid phase (g/L)
*D*: diffusion coefficient (m^2^/s)
*k*: coefficient of Chrastil’s equation (-)
*k_f_*: mass transfer coefficient in liquid phase (m/s)
*k_s_*: mass transfer coefficient in solid phase (m/s)
*m*: mass (g)
*p*: pressure (Pa)
*q_m_*: mass transfer rate (s^−1^)
*r*: fraction of less accessible solute (-)
*Re*: Reynolds number (-)
*Sc*: Schmidt number (-)
*t*: time (s)
*t_CER_*: end-time of CER stage (s)
*t_FER_*: end-time of FER stage (s)
*T*: temperature (K)
*u*: flow speed (m/s)
*v*: volume (L)
*W*: parameter of BIC model (-)
*x*: extraction yield (g/g)
*x_0_*: total content of analyte in matrix (g/g)
*y^*^*: solubility (g/g)
*Z*: parameter of BIC model (-)
*Z_W_*: parameter of BIC model (-)
*ε*: bed porosity (-)
*μ*: dynamic viscosity (Pa·s)
